# Supplementary material for: Construction of model animals to explore intestinal microbiome for detection of breast cancer
Source: PLoS One. 2023 May 17;18(5):e0280971. doi: 10.1371/journal.pone.0280971 (PMC10191350; doi:10.1371/journal.pone.0280971)
Supplement: Attachment — Submitted filename: Response to Reviewers.docx [file pone.0280971.s001.docx]

To the academic editor:

1. We have revised our manuscript to PLOS ONE's style requirements and highlight them in papers.
2. We report additional details in your Methods section regarding animal care, as the editorial guidelines at this time:

1) We provide details of animal welfare including shelter, food, water, and environmental enrichment in line 98.

2) We used 100 mg/kg pentobarbital to administer intraperitoneal injections to euthanasia of the mice that minimize animal suffering and distress in line 102.

3) In our experiments, the maximum size of tumors does not grow to more than 100 mm^3^, and we provide this in line 102.

4) We describe the post-operative and post tumor implantation care received by the animals, including the frequency of monitoring and the criteria used to assess animal health and well-being in line 100.

3. We have corrected the grant information in the “Funding Information” and “Financial Disclosure”.

4. We state that “This study was supported by grants from the Applied Basic Research Programs of Science and Technology Commission Foundation of Guangdong Province (No. 2022A1515012602) and Foundation of Guangdong Educational Committee for Youths (No. 2019KQNCX150). The funders had no role in study design, data collection and analysis, decision to publish, or preparation of the manuscript.” And we include this amended Role of Funder statement in our cover letter.
5. We have deleted funding information in the Acknowledgments section and include your amended statements within your cover letter.
6. We have completed our Competing Interests on the online submission form to state any Competing Interests as "The authors have declared that no competing interests exist." And we include this amended Role of Funder statement in our cover letter.
7. We have changed our Data Availability provide information statement to be released, and the Bioproject was released and include your amended statements within your cover letter.
8. I ensure that I have an ORCID Id (0009-0008-2283-1733) and that it is validated in Editorial Manager.
9. We have not found the institution and department of Tianshu Cai and Weiwei Li in the submission part, could you please add their information? Their institution is Huizhou Health Sciences Polytechnic and the department is Department of Pharmacy and Laboratory.
10. We have reviewed our reference list to ensure that it is complete and correct, and we have not cited papers that have been retracted.

To reviewer1:

1. We considered that the limitations of their study were that the variation in the abundance of bacterial species found in this study is slightly different from the results of other studies, which may be caused by the differences in the environment of human clinical samples or the host of different species. Laboratory mouse models with the same culture environment can overcome this, and if the clinical samples are combined with this project, it will have better practical significance., and we add this in the discussion in line 254.

To reviewer2:

1. We have checked grammar in the manuscript again.
2. We have described more about the breast cancer model and methods for generating the model for breast cancer studies in line 95.
3. We also added more about the way our characterize various microbes in the gut in line 227.
